# Latest approaches on the management of OA in humans

**DOI:** 10.1186/1471-2474-16-S1-S4

**Published:** 2015-12-01

**Authors:** Nidhi Sofat

**Affiliations:** 1 St Georges, University of London, UK

## 

The Sofat laboratory currently investigates the mechanisms responsible for pain and tissue damage in arthritis. Clinical studies include ‘Pain management in osteoarthritis using centrally acting analgesics’ (http://public.ukcrn.org.uk/search/StudyDetail.aspx?StudyID=12175) funded by the Rosetrees Trust. This clinical trial is testing whether drugs that inhibit pain processing pathways in the brain can help with pain in the hand caused by osteoarthritis. Other clinical studies include the ‘Pain Perception in Osteoarthritis’, or PAPO study, investigating tissue damage and pain in people undergoing knee replacement surgery for osteoarthritis. Both studies are on the UK NIHR portfolio of network-adopted studies.

Our laboratory has a translational approach, with techniques including magnetic resonance imaging (MRI) to decipher regions of damage in affected joints and evaluating which brain regions are activated by arthritis pain. We also use non-invasive quantitative sensory testing (QST) methods to identify pain pathways. QST identifies sensation and pain thresholds by stimulating the skin.

Our laboratory research uses a basic science mechanistic approach to investigate the influence of endogenous damage-associated molecular patterns (DAMPs) in driving chronic inflammation in joints. Molecules of interest include interaction of the DAMPs tenascin-C and fibronectin with oral pathogens.

